# Correspondence

**Published:** 1912-06

**Authors:** J. M. Higgins

**Affiliations:** Arizpe, Sonora, Mex.


### CORRESPONDENCE

Arizpe, Sonora, Mex., May 4, 1912.

Buffalo Medical Journal, Buffalo, N. Y.

Dear Dr.—I don't expect that this will interest you much but I wish to protest in the columns of your journal against the false statements of the U. S. press in regard to our trouble here in Mexico, especially here in Sonora.

I have lived in Mexico now for over a year and have always been treated with courtesy by the Mexican people, both rebels and federals. I have met and talked with Americans from all over the republic, even from the danger zone in Chihuahua and the lower states and each and every one of them reports that he has been treated kindly by the Mexican people and brands the border press as falsifiers.

We will take the state of Sonora as a whole and there are not over thirty so-called revolutionists and it is only a matter of a few days till they will be captured. The Yaqui Indians are loyal to the government to a man and all reports to the contrary are false.

Madero has made an able executive and if the U. S. press, especially the border press, would confine itself to the truth it would only be a few weeks till we would have peace all over the republic. Yours respectfully,

DR. J. M. HIGGINS.

